# Evaluation of Root Canal Cleaning and Irrigant Penetration Using Different Irrigation Protocols: A Combined SEM and Single‐Tooth Micro‐CT Study

**DOI:** 10.1002/cre2.70175

**Published:** 2025-07-17

**Authors:** Alfredo Iandolo, Niccolò Giuseppe Armogida, Davide Mancino, Gianrico Spagnuolo, Mariangela Cernera, Dina Abdellatif

**Affiliations:** ^1^ Centre Hospitalier Universitaire, CHU Besançon France; ^2^ Laboratoire Sinergies EA 4662 University of Marie et Louis Pasteur Besançon France; ^3^ Department of Neuroscience, Reproductive Sciences and Dentistry University of Naples Federico II Naples Italy; ^4^ Faculty of Dental Surgery, Federation of Medicine Translational of Strasbourg and Federation of Materials and Nanoscience of Alsace University of Strasbourg France

**Keywords:** endodontics, heating activation, root canal irrigants, smear layer removal, sonic irrigation, ultrasonic irrigation

## Abstract

**Objectives:**

This study evaluated the effectiveness of different irrigation techniques in removing debris and smear layers and in improving irrigant penetration within root canals. Particular attention was given to the 3D Cleaning technique, which combines ultrasonic activation with internal heating of sodium hypochlorite.

**Material and Methods:**

Fifty‐one single‐rooted human mandibular premolars were used. Group A (*n* = 50) was divided into five subgroups (*n* = 10 each), each receiving a different irrigation protocol (control, syringe irrigation, sonic activation, ultrasonic activation, and 3D Cleaning). Scanning electron microscopy (SEM) at ×1000 magnification was used to evaluate debris and smear layer removal based on a validated scoring system. Group B (*n* = 1) consisted of a single premolar with a lateral canal in the apical third, examined using microcomputed tomography (micro‐CT). The same tooth underwent all five irrigation protocols in sequence, with radiopaque irrigant penetration evaluated after each and confirmed through sequential micro‐CT scans. A power analysis confirmed the adequacy of sample size (*n* = 50; α = 0.05, power = 80%). Non‐parametric tests were used, including Kruskal–Wallis ANOVA for intergroup comparisons, followed by Mann–Whitney *U* tests with Bonferroni correction for post hoc pairwise analysis (*p* < 0.05 considered significant).

**Results:**

The 3D Cleaning technique (ultrasonic activation + internal heating) achieved the lowest debris and smear layer scores and the highest irrigant penetration depth (~0.6 mm) into the lateral canal. The control group showed no penetration and the highest debris scores. Conventional syringe and sonic activation showed moderate cleaning and penetration (0.2–0.3 mm), while ultrasonic activation alone showed slightly better results (~0.4 mm).

**Conclusion:**

The 3D Cleaning technique demonstrated superior effectiveness in root canal cleaning and irrigant penetration compared to conventional irrigation methods. These results support the clinical relevance of heating‐activated irrigation protocols to enhance root canal disinfection.

## 1 Introduction

The cleaning phase constitutes an essential aspect of root canal treatment, facilitating the disinfection of the intricate endodontic system through the application of irrigants (Gomes et al. 2023). Evidence indicates that the shaping phase generates a smear layer, which adheres randomly to the root canal walls, exhibiting a thickness ranging from 2 to 5 μm (de Oliveira Neto et al. 2023; Violich and Chandler 2010). This smear layer primarily comprises a crystalline structure that includes remnants of pulp, dentinal debris, microorganisms, and their metabolic byproducts (de Oliveira Neto et al. 2023). In the presence of infection, the smear layer becomes contaminated, harboring bacteria within the dentinal tubules, which impedes the efficient penetration of irrigants and intracanal medicaments (Paixão et al. 2022a). Furthermore, the smear layer exacerbates microleakage at both coronal and apical ends by interfering with the sealing capabilities of root canal sealers (Rao et al. 2023). Consequently, removing the smear layer is critical for ensuring adequate disinfection within the primary root canal and its lateral anatomies, such as lateral canals, loops, microconnections, isthmuses, and dentinal tubules.

Research has confirmed that the smear layer formation is most pronounced in the apical third of the root canal, rendering its removal particularly challenging due to the complex anatomy involved (Nangia et al. 2020). Data obtained from a systematic review suggest that eliminating the smear layer correlates with enhanced clinical outcomes in root canal therapy (Fortea et al. 2024). Previous studies have underscored the significance of chemo‐mechanical debridement techniques in effectively removing the smear layer's inorganic and organic constituents from the root canal system. The standard protocol for smear layer removal involves the combined application of sodium hypochlorite (NaOCl) and ethylenediaminetetraacetic acid (EDTA), each administered for 1 min (Fortea et al. 2024). However, various factors, including the efficiency of the process, significantly influence the debridement phase, with irrigant activation playing a pivotal role (Uroz‐Torres et al. 2010).

Current methodologies for irrigant activation encompass ultrasonic activation, sonic file activation, laser activation, and intracanal heating activation (Susila 2019). Ultrasonic activation utilizes high‐frequency ultrasonic tips (25–40 kHz) to create acoustic streaming, thereby enhancing irrigant flow, removing debris, and augmenting antibacterial effectiveness within the root canal (Boutsioukis and Arias‐Moliz 2022). Conversely, sonic file activation employs oscillating sonic files at lower frequencies (1–6 kHz) to agitate irrigants, facilitating the removal of the smear layer, albeit with reduced efficiency (Paixão et al. 2022b). Laser activation employs high‐energy laser pulses to generate photoacoustic shock waves, significantly enhancing both irrigant penetration and disinfection (Usta et al. 2024). However, its high cost and the associated risk of apical extrusion limit its clinical applications (Šnjarić et al. 2025). Intracanal heating activation involves directly heating NaOCl within the root canal, improving its tissue dissolution, antimicrobial properties, and flow characteristics, thus enhancing cleaning efficacy (Iandolo et al. 2021; Tartari et al. 2021).

Recent investigations have introduced a novel root canal irrigation technique known as 3D Cleaning, proposed as an alternative to traditional syringe needle irrigation (Pantaleo et al. 2022). This technique integrates ultrasonic activation of heated NaOCl within the root canal system. Reported benefits of 3D Cleaning include lower costs compared to laser‐based activation, enhanced pulp tissue dissolution, improved antibacterial efficacy, and deeper penetration of irrigants for optimized disinfection.

The 3D Cleaning technique involves controlled intracanal heating of sodium hypochlorite using a heat source (System‐B Heat Source; Analytic Endodontics, Orange, CA, USA) set at 180°C, with an X‐fine tip (30/04) positioned 3 mm short of the working length. The tip was activated for 8 s without contacting the canal walls. During this phase, the temperature of sodium hypochlorite reached approximately 80°C, well below its boiling point (Pantaleo et al. 2022; Iandolo et al. 2017). This was followed by 20 s of ultrasonic activation. It is expected that the temperature of the solution decreases during the ultrasonic phase.

This study aims to assess root canal cleanliness following the 3D Cleaning approach through scanning electron microscopy (SEM) and micro‐CT analysis to compare its effectiveness with conventional irrigant activation methods. The null hypothesis for this study posits that there will be no statistically significant difference in debris and smear layer formation between the 3D Cleaning technique and other irrigation methodologies. Additionally, it is anticipated that no significant differences will be observed in the depth of irrigant penetration among the various irrigation techniques evaluated.

## 2 Materials and Methods

The study was conducted according to the guidelines of the Declaration of Helsinki and approved by the Institutional Review Board: protocol code CE‐2025‐16, University of Strasbourg, France.

Extracted mandibular single‐rooted premolars (*n* = 51) were chosen for this study. These mandibular premolars were extracted as part of an orthodontic treatment plan and were irrelevant to the current work. Informed consent was acquired from the patients.

### 2.1 Sample Distribution and Study Design

The study was divided into two parts: **Group A** for SEM analysis and **Group B** for Micro‐CT analysis.

**Group A** included 50 extracted single‐rooted mandibular premolars, which were randomly assigned to five experimental subgroups (*n* = 10 per group), each subjected to a different irrigation protocol. These teeth were decoronated, instrumented, and processed for SEM imaging by longitudinal sectioning to evaluate debris and smear layer removal.

**Group B** consisted of one additional mandibular premolar, selected based on the presence of a lateral canal identified via micro‐CT scanning. This same tooth was subjected sequentially to the five irrigation protocols used in Group A. After each irrigation protocol and contrast medium application, a micro‐CT scan was performed to measure the depth of irrigant penetration into the lateral canal. To prevent bias, the radiopaque contrast solution (Hypaque) was completely aspirated, and its removal was confirmed before each subsequent scan.

This design allowed for standardization and direct comparison of irrigant penetration across different activation methods within the same anatomical conditions, while SEM and Micro‐CT analyses were conducted on separate specimens to avoid methodological interference.

Exclusion criteria were fracture, crack, resorption, periapical lesion, previously root canal‐treated tooth, history of trauma, immature apex, and calcification. Inclusion criteria were closed apices, normal anatomy, single root canals, curvatures less than 10°, and sound teeth extracted for orthodontic purposes.

The periodontal tissues on the external surface of the teeth were removed. The specimens were preserved in separate vials containing 10% buffered formalin and stored at 15°C for up to 30 days until the experiment (Iandolo et al. 2021).

Before the experiment started, all teeth were randomly assigned to groups of 10 specimens, each using a sequence generated by Random Allocation Software 2.0. This guaranteed allocation concealment from investigators and ensured the experiment's fairness.

#### 2.1.1 Root Canal Preparation

The teeth were decoronated at the cementoenamel junction to obtain roots of standardized length (18 mm). A size 10 K‐file was inserted into each canal until it was visible from the apical foramen. The working length was verified by subtracting 0.5 mm from this measurement.

The apices were closed using wax to mimic the periodontal ligament. The canals were shaped with nickel‐titanium rotary instruments (Hyflex EDM, Coltene/Whaldedent Inc., Cuyahoga Falls, USA). Only the 10/0.05 and 20/0.05 instruments of Hyflex EDM were used to prepare the canals to the full working length. This step was done intentionally to ensure minimal preparation of the root canals.

During canal instrumentation, irrigation was performed with 3% NaOCl using a side‐vented 30 G needle in a syringe.

5 mL of NaOCl was used for every tooth and refreshed every minute. The root canals were then rinsed with sterile saline.

At the end of the shaping phase, different protocols of irrigant activation of NaOCl were used.

Throughout all experiments, the roots of the premolars were maintained at 36.8°C using a thermostat with real‐time display and control capabilities (Vevor, SH‐3ABEII, Shanghai, China). This temperature was selected to simulate physiological conditions, ensuring clinical relevance. A thermocouple was employed to verify the liquid temperature, guaranteeing accuracy within ±0.1°C.

Group (A1) saline solution (control): an endodontic needle, side‐vented 30 G, reached 2 mm shorter than the working length, and 5 mL of saline was used. 2 mL of saline solution was used as the final flush, and paper points were used to dry the canals.

Group (A2): 3 mL of 17% EDTA for 1 min was used, followed by 3 mL of sterile saline. A side‐vented 30 G needle delivered the irrigants inside the canal 2 mm shorter than the working length. Finally, traditional irrigation was performed. An endodontic needle reached 2 mm shorter than the working length, and 5 mL of NaOCl was used. 2 mL of saline solution was used as the final flush, and paper points were used to dry the canals.

Group (A3): 3 mL of 17% EDTA for 1 min was used, followed by 3 mL of sterile saline. A side‐vented 30 G needle delivered the irrigants 2 mm shorter than the working length. Finally, the sonic activation was performed: NaOCl was delivered 2 mm shorter than the working length.

The eddy tip (VDW GmbH, Munich, Germany) was 3 mm shorter than the working length; it was not in contact with dentinal walls and was activated for 20 s. The tip used for sonic activation was 0.20 mm. This activation procedure was repeated three times, and NaOCl was refreshed with a new solution at each cycle. A total of 6 mL of NaOCl was used. 2 mL of saline solution was used as the final flush, and paper points were used to dry the canals.

Group (A4): 3 mL of 17% EDTA for 1 min was used, followed by 3 mL of sterile saline. As in the other groups, a side‐vented 30 G needle delivered the irrigant 2 mm shorter than the working length. Finally, the ultrasonic activation was performed: NaOCl was delivered 2 mm shorter than the working length.

Twenty seconds of ultrasonic activation using the Ultra Smart AI (Coxo, Fushan, China) were performed. The tip for ultrasonic activation was 0.25 mm, 3 mm shorter than the working length. This activation procedure was repeated three times, and NaOCl was refreshed with a new solution at each cycle. A total of 6 mL of NaOCl was used. 2 mL of saline solution was used as the final flush, and paper points were used to dry the canals.

Group (A5): 3 mL of 17% EDTA for 1 min was used, followed by 3 mL of sterile saline. Also, this group used a side‐vented 30 G needle to deliver the irrigant 2 mm shorter than the working length. Finally, internal heating combined with ultrasonic activation was performed: NaOCl was delivered 2 mm shorter than the working length.

System‐B Heat Source (Analytic Endodontics, Orange, Ca, USA) set at 180°C was used with an X‐fine tip (30/04) at 3 mm shorter than the working length; the tip was not in contact with dentinal walls and was activated for 8 S. Followed 20 s of ultrasonic activation using the Ultra Smart AI (Coxo, Fushan, China). The tip used for ultrasonic activation was 0.25 mm. used at 3 mm shorter than the working length. This activation procedure was repeated three times, and NaOCl was refreshed with a new solution at each cycle. A total of 6 mL of NaOCl was used. 2 mL of saline solution was used as the final flush, and paper points were used to dry the canals.

The teeth in Group A were decorated at the cement–enamel junction (CEJ) and examined using scanning electron microscopy (SEM). Debris and Smear layers were viewed at a magnification of ×1000 and scored.

#### 2.1.2 Group A

Two longitudinal grooves were prepared on each root's palatal/lingual and buccal surfaces with a diamond bur and a high‐speed and water‐cooling handpiece to facilitate vertical splitting. After canal preparation, the specimens were immersed in liquid nitrogen and split longitudinally into two halves with a stainless steel chisel. The sections were then prepared for SEM analysis: They were allowed to air‐dry overnight in a desiccator at room temperature, sputter‐coated with gold, and prepared for SEM analysis (EVO MA 10 Carl Zeiss SMT AG, Germany).

**Figure 1:**
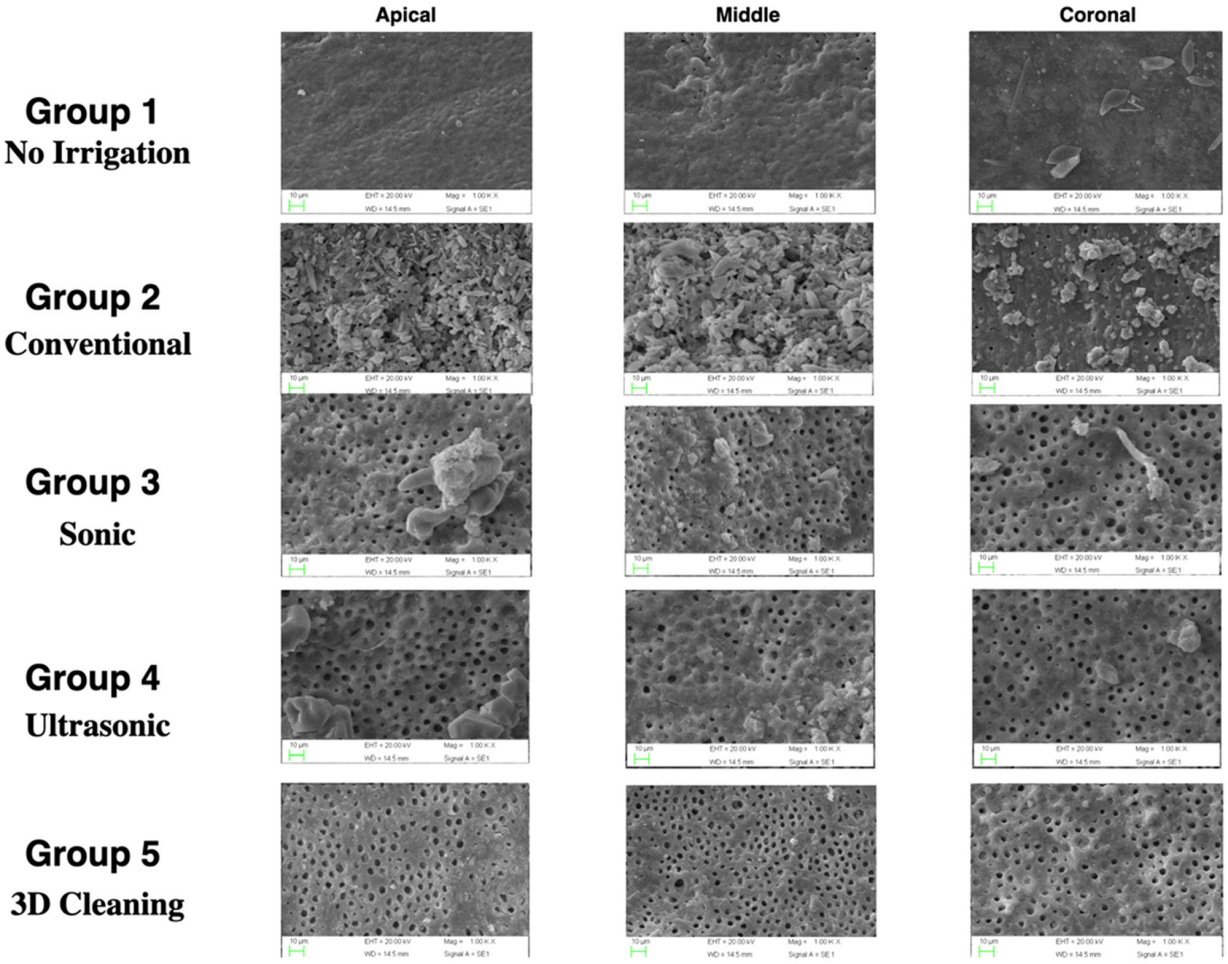
Representative samples of scanning electron microscope images of the coronal, middle and apical third following irrigation of the control group (Group 1), traditional irrigation group (Group 2), sonic activation (Group 3), ultrasonic activation (Group 4) and Internal heating combined with ultrasonic activation (Group 5) (1000× magnification).

Scanning electron microscopy (SEM) images were obtained at a magnification of ×1000 (Figure 1).

A total of 30 photomicrographs were captured, with 10 images taken from each third of the root canal (coronal, middle, and apical). The selected areas were standardized across samples to represent equivalent anatomical regions. Three calibrated and blinded evaluators independently assessed the presence or absence of debris and smear layer on the canal wall surfaces. To ensure consistency among evaluators, inter‐examiner agreement was calculated using Fleiss' kappa, which showed substantial agreement (κ = 0.73). The rating system used was proposed by Hulsmann et al (Rödig et al. 2007), and the criteria for the scoring were reported as follows.

##### 2.1.2.1 Scores of the Debris

Score 1: Clean root canal walls with only a few small debris particles.

Score 2: Few small agglomerations of debris.

Score 3: Many agglomerations of debris covering < 50% of the root canal walls.

Score 4: > 50% of the root canal walls are covered by debris.

Score 5: Complete or nearly complete root canal walls covered by debris.

##### 2.1.2.2 Scores of the Smear Layer

Score 1: No smear layer, orifices of dentinal tubules open.

Score 2: Small amount of smear layer, some dentinal tubules open.

Score 3: Homogenous smear layer covering the root canal walls, only a few dentinal tubules open.

Score 4: Complete root canal wall covered by a homogenous smear layer, no open dentinal tubules.

Score 5: The heavy, homogenous smear layer covers the entire root canal walls.

#### 2.1.3 Group B

The tooth in Group B was examined using microcomputed tomography (Micro‐CT). In this group, the penetration depth of the irrigant was evaluated using various irrigation techniques.

A mandibular premolar was selected based on a micro‐CT scan, presenting a lateral canal in the apical third, located 4 mm from the working length. The shaping phase was performed similarly to the samples that had been prepared previously. Hypaque (Diatrizoate sodium, MyBioSource Inc., San Diego, CA, USA, according to manufacturer's specifications, Hypaque presents a viscosity of 2.0–2.5 cP, surface tension of 70–75 mN/m, and specific gravity of 1.21 g/cm³ at room temperature), a radiopaque solution with viscosity, surface tension, and specific gravity comparable to 5.25% sodium hypochlorite (NaOCl), was employed as an irrigant in place of NaOCl (Schoeffel 2007; Bronnec et al. 2010).

Additionally, the standardization was endured by using the same premolar for all irrigation protocols. This approach ensured methodological consistency by allowing direct comparison of the various irrigation techniques under identical anatomical and experimental conditions, eliminating the need for additional samples.

### 2.2 Irrigation Protocol for Micro‐CT Evaluation

The irrigation protocols applied in Group B mirrored those used in Group A. The same five irrigation techniques (control with no activation, syringe irrigation, sonic activation, ultrasonic activation, and 3D Cleaning) were performed sequentially on a single premolar with a lateral canal. Each protocol included the same volumes, irrigant types, and activation times as described for Group A.

In the first experiment (*n* = 10), Group B‐1, the cleaning protocol was conducted identically to Group A1 from the previous experiment. Subsequently, an X‐ray scan was performed using the Micro‐CT scanner (SkyScan 1072, SkyScan, Belgium). Scanning parameters included a source voltage of 100 kV, a current of 98 µA, and a magnification factor of ×15, achieving a volumetric resolution of 19.1 µm × 19.1 µm × 38.0 µm. The SKYSCAN 1072 measurement software was employed to quantify the penetration depth of the radiopaque irrigant within the lateral canal.

In the second experiment (*n* = 10), Group B‐2, the cleaning protocol corresponded to that of Group A2 from the prior study, followed by an X‐ray scan.

In the third experiment (*n* = 10), Group B‐3, the cleaning protocol matched Group A3 from the previous study, followed by an X‐ray scan.

In the fourth experiment (*n* = 10), Group B‐4, the cleaning protocol was consistent with Group A4 from the prior study, followed by an X‐ray scan.

In the fifth experiment (*n* = 10), Group B‐5, the cleaning protocol replicated Group A5 from the previous study, followed by an X‐ray scan.

**Figure 2:**
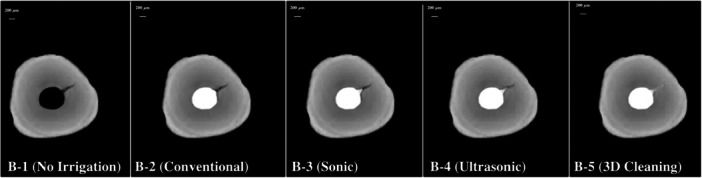
Representative samples of micro‐CT images of the different groups: B‐1 (without irrigant), B‐2 (traditional irrigation), B‐3 (sonic activation), B‐4 (ultrasonic activation), and B‐5 (internal heating combined with ultrasonic activation). These images illustrate the penetration of the radiopaque irrigant into the lateral canal, depending on the irrigation technique used.

For all groups (B‐1, B‐2, B‐3, B‐4, and B‐5), 10 tests per group were conducted, resulting in 50 tests overall. After each test, the sample underwent a micro‐CT scan. The radiopaque irrigant (Hypaque) was fully aspirated using an endodontic needle (Surgi Tip Endo, Roeko, Coltene). No additional rinsing or cleaning procedures were performed. A subsequent scan was performed to confirm the complete removal of the contrast medium before proceeding with the next experimental group, thereby ensuring that each group started under the same baseline conditions. Additionally, the sequence of the experimental groups was randomized to prevent any systematic bias potentially arising from repeated irrigations or residual solution accumulation in lateral anatomical complexities. The SKYSCAN 1072 measurement software was utilized consistently to measure the penetration depth of the radiopaque irrigant within the lateral canal across all experimental groups and tests (Figure 2).

### 2.3 Statistical Analysis and Sample Size Calculation

#### 2.3.1 Sample Size Calculation

The sample size was determined using G*Power Software, with an effect size of 0.4 and a significance level (α) of 0.05. To achieve a statistical power of 80%, 50 samples were required, 10 samples per group.

Statistical analysis was performed using Stata 12.0 software (Stata, College Station, Texas, USA). Debris and smear layer scores were recorded separately for all groups. Descriptive statistics were calculated for ordinal data in each experimental group, including the median, minimum, and maximum values.

A nonparametric analysis of variance (Kruskal–Wallis ANOVA) was used to compare the debris and smear layer scores among the different irrigation techniques. When significant differences were detected, Mann–Whitney *U* tests were used for post hoc pairwise comparisons, and a Bonferroni correction was applied to control for the risk of Type I errors associated with multiple comparisons. A *p*‐value < 0.05 was considered statistically significant for all tests.

For Group B, the penetration depth of the irrigant was measured using Micro‐CT imaging, and the values were analyzed descriptively.

## 3 Results

### 3.1 Debris and Smear Layer Scores (Group A – Figure 3)

**Figure 3:**
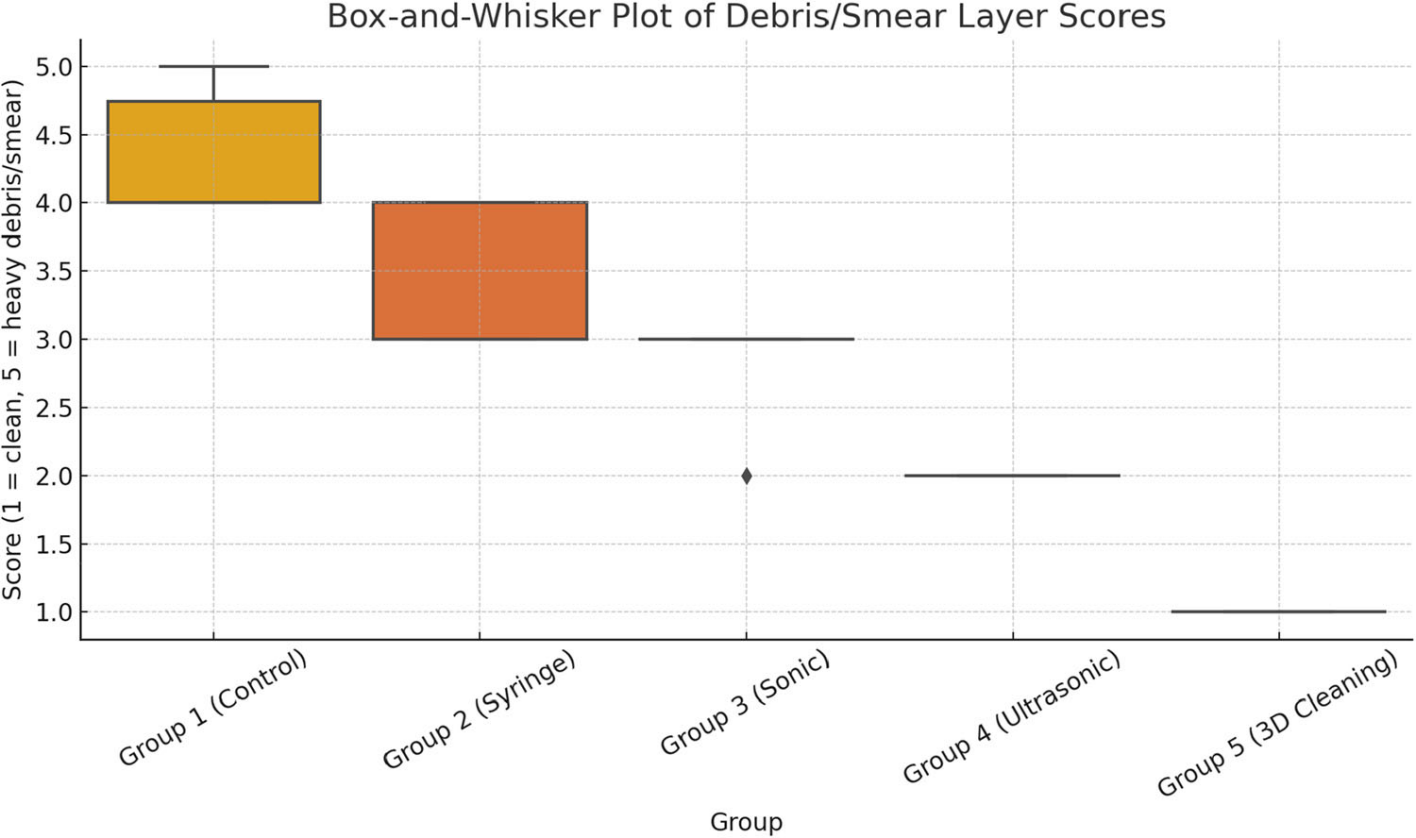
Group A: Debris and Smear Layer Scores – Box‐and‐whisker plot showing the distribution of debris and smear layer scores for each irrigation technique. Lower scores indicate cleaner root canal walls. Group 5 (Ultrasonic Activation + Internal Heating) exhibited the lowest scores, representing the most effective cleaning, while Group 1 (Control) showed the highest scores, indicating poor cleaning efficacy. The plot illustrates median values, interquartile ranges, and potential outliers, providing a non‐parametric representation suitable for ordinal data.

Statistical analysis revealed significant differences in debris and smear layer scores among the groups (*p* < 0.05, Kruskal–Wallis ANOVA). Post hoc comparisons using the Mann–Whitney *U* test showed that Group 5 (Ultrasonic Activation + Internal Heating) achieved the lowest median scores, indicating the most effective canal cleaning, while Group 1 (Control) consistently had the highest scores, confirming no cleaning effect.

- Group 5 demonstrated significantly lower debris and smear layer scores compared to Groups 1–4 (*p* < 0.05).
- A progressive improvement in cleaning efficacy was observed across the groups, with Group 2 (Syringe Irrigation) showing modest improvement and Group 4 (Ultrasonic Activation) improving the results.
- Scores were consistently higher in the apical third compared to the coronal and middle thirds across all groups, indicating the inherent difficulty of cleaning the apical region.
- The standard deviation for debris scores was highest in Groups 2 and 3, indicating variability in cleaning effectiveness across samples.
- For smear layer scores, a similar trend was observed:
- Group 5 had the lowest median scores, with minimal variability (SD < 1).
- Group 1 exhibited the highest scores, with minimal deviation due to a consistent lack of cleaning.

### 3.2 Irrigant Penetration Depth (Group B – Figure 4)

**Figure 4:**
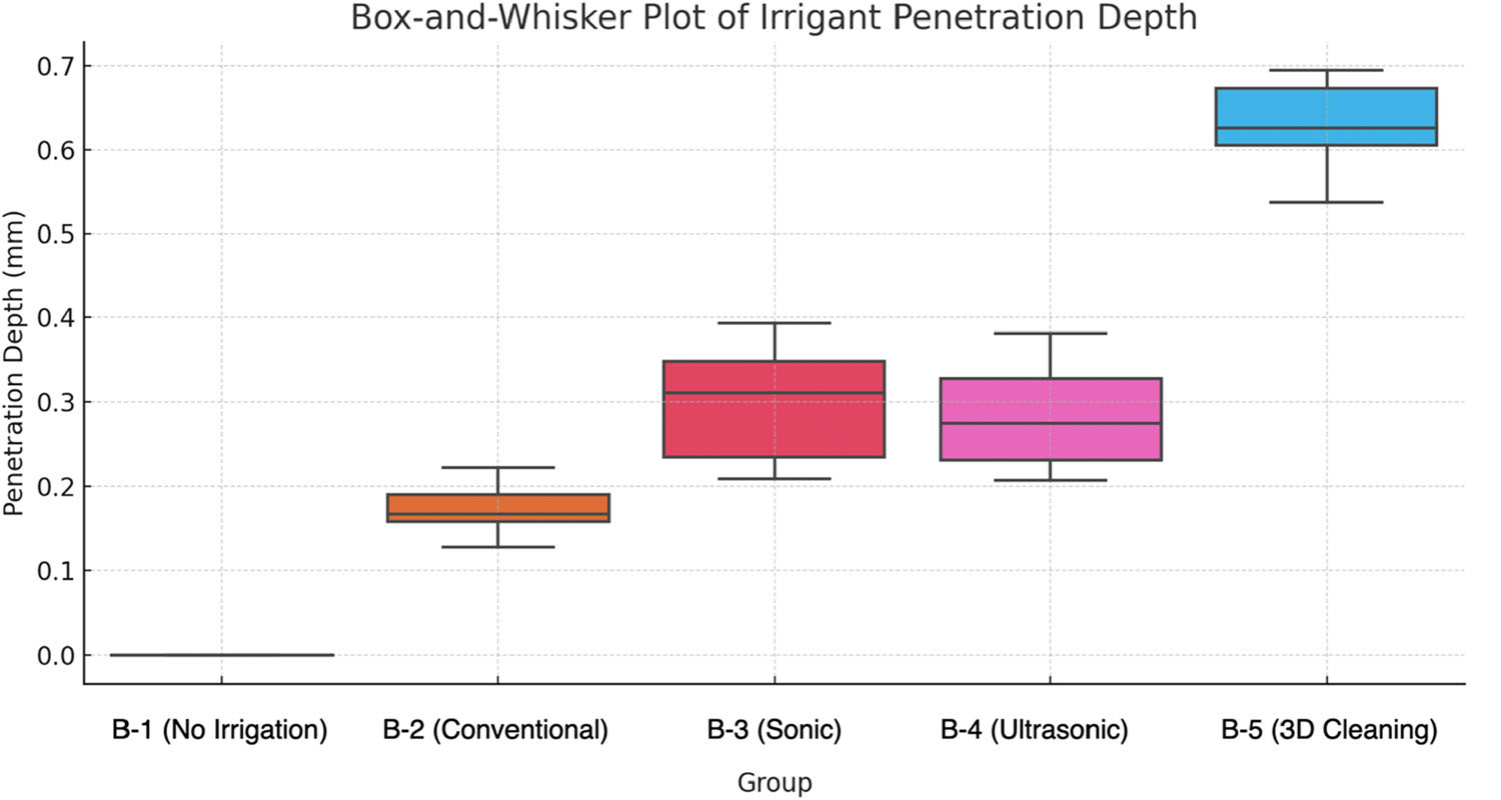
Group B: Irrigant penetration depth, box‐and‐whisker plot showing the distribution of irrigant penetration depth (mm) into the lateral canal for each irrigation technique. B‐5 (Ultrasonic Activation + Internal Heating) demonstrated the greatest median penetration (~0.6 mm), while B‐1 (No Irrigation) showed no penetration (0 mm). The plot reflects the median, interquartile range, and variability across samples, and is more suitable for non‐parametric data than bar charts.

Micro‐CT analysis of the penetration depth demonstrated significant differences among the irrigation techniques. Descriptive statistics showed a clear trend:

- B‐1 (No Irrigation): No penetration (0 mm).
- B‐2 (Conventional Irrigation): Limited penetration (0.1–0.3 mm).
- B‐3 (Sonic Activation): Moderate penetration (0.2–0.4 mm).
- B‐4 (Ultrasonic Activation): Improved penetration (0.2–0.4 mm).
- B‐5 (Ultrasonic Activation + Internal Heating): The deepest penetration (0.5–0.7 mm), significantly higher than other groups (*p* < 0.05).
- The penetration depth increased with more advanced irrigation techniques:
  ◦ B‐5 (Ultrasonic Activation + Heating): Achieved the highest median penetration (0.6 mm), with values ranging from 0.5 to 0.7 mm and a standard deviation of 0.09 mm.
  ◦ B‐1 (No Irrigation): Recorded a median penetration of 0 mm, with no variability.
  ◦ B‐2 (Conventional Irrigation) and B‐3 (Sonic Activation) demonstrated moderate penetration depths (median: 0.2–0.3 mm) with some variability.

#### 3.2.1 Key Observations

- The results demonstrate that Ultrasonic Activation with Internal Heating (Group 5/B‐5) was the most effective technique for both cleaning and irrigant penetration, achieving statistically significant improvements over other techniques.
- Traditional syringe irrigation (Group 2/B‐2) was significantly less effective than advanced techniques.
- The apical third consistently showed higher debris and smear layer scores and lower penetration depths, emphasizing the challenge of effectively cleaning this region.

These findings highlight the importance of using advanced irrigation techniques, particularly Ultrasonic Activation with Heating, to achieve optimal cleaning and irrigant delivery in endodontic procedures.

### 3.3 Effectiveness of 3D Cleaning Method

- **Smear Layer Removal:** The 3D Cleaning method (ultrasound + heating) shows a statistically significant improvement in smear layer removal compared to the control and other tested techniques. The combined physical (ultrasound‐induced cavitation and acoustic streaming) and thermal effects enhance smear layer disruption and detachment from dentinal tubules.
- **Debris Removal:** Debris removal is also significantly enhanced by the 3D Cleaning method. Heating softens organic remnants and reduces the viscosity of irrigants, improving their penetration and flushing ability, while ultrasonic agitation ensures better irrigant circulation and physical displacement of particles.

### 3.4 Mechanism of Enhanced Cleaning by Heating

The synergistic effect of heating and ultrasound can be attributed to:

- **Reduced Surface Tension and Viscosity:** Heated irrigants (e.g., NaOCl) exhibit increased wettability and flow, allowing deeper penetration into dentinal tubules and canal irregularities.
- **Enhanced Chemical Reactivity:** Higher temperatures accelerate the dissolution of organic tissues by NaOCl and increase its reactivity.
- **Improved Acoustic Streaming:** Heat may slightly increase fluid motion caused by ultrasonic agitation, amplifying the mechanical cleaning effect.

## 4 Discussions

The success of endodontic therapy relies on effective cleaning, shaping, and sealing of the root canal system (Gulabivala and Ng 2023). The primary aim of root canal treatment is to eliminate or reduce bacterial contamination while preventing reinfection (Babeer et al. 2024). However, mechanical instrumentation alone is insufficient for removing all debris and bacteria, as modern Ni‐Ti rotary systems create a smear layer that adheres to the canal walls (Versiani et al. 2023). Therefore, irrigating solutions are essential for disinfecting the root canal system and enhancing the removal of debris and the smear layer (Gomes et al. 2023).

Sodium hypochlorite (NaOCl) is the most commonly used endodontic irrigant due to its antimicrobial properties, tissue dissolution capability, lubrication, and debris‐flushing ability (Gomes et al. 2023; Zollinger et al. 2019). However, NaOCl by itself cannot effectively remove the smear layer, necessitating the use of ethylenediaminetetraacetic acid (EDTA) as a chelating agent (Herrera et al. 2017). Various irrigant activation techniques have been developed to improve penetration and effectiveness, including ultrasonic activation, sonic activation, intracanal heating, and laser techniques (Gomes et al. 2023; Iandolo et al. 2021).

This study evaluated the efficacy of different irrigant activation techniques, with a particular emphasis on ultrasonic activation combined with internal heating. The null hypothesis was rejected, as statistically significant differences were found in the removal of debris and the smear layer between the 3D cleaning technique and other irrigation techniques. Moreover, significant differences were also observed in irrigant penetration depth among the various irrigation protocols.

The improved penetration of the irrigant into lateral canals observed in the 3D Cleaning group can be attributed to the controlled intracanal heating of sodium hypochlorite. Heating significantly reduces the viscosity of NaOCl, enhancing its flow characteristics and allowing it to navigate more effectively into microanatomical areas such as lateral canals, loops, and isthmuses. When combined with ultrasonic activation, this thermal modification is further potentiated by the mechanical effects of acoustic streaming and cavitation, resulting in superior irrigant penetration. These findings are supported by the study of Iandolo et al. (2021), which demonstrated enhanced pulp tissue dissolution in an isthmus model using heated NaOCl in conjunction with ultrasonic activation (Iandolo et al. 2021).

The results indicated that ultrasonic activation with internal heating provided the most effective removal of debris and the smear layer, along with significantly greater irrigant penetration, especially in the apical third.

The results of our study demonstrated that ultrasonic activation combined with internal heating (Group B‐F) achieved the highest irrigant penetration into lateral branches, with median values around 0.6 mm. These findings are consistent with the literature emphasizing the importance of advanced activation protocols for improved irrigant delivery.

A recent study (Fidan and Erdemir 2023) evaluating the effectiveness of XP‐endo Finisher, EDDY, and laser‐based systems (Nd:YAG and Er:YAG) on simulated lateral canals reported that Er:YAG laser activation provided effective contrast solution penetration, particularly at the coronal and middle thirds. In that study, penetration depth progressively decreased toward the apical third, which aligns with our findings showing reduced penetration in deeper canal regions. While laser activation demonstrated effective results, its cost, technique sensitivity, and risk of apical extrusion limit its widespread clinical use. In contrast, the 3D Cleaning protocol used in our study offers a practical and accessible alternative, achieving comparable effectiveness in irrigant penetration without the drawbacks associated with laser‐based systems.

These findings are consistent with previous studies that demonstrated the benefits of heated NaOCl in enhancing its tissue dissolution capacity (Usta et al. 2024; Damade et al. 2020). A study by Iandolo et al. (2018) showed that the internal heating technique achieved good smear layer and debris removal even in the absence of EDTA. Similarly, our research underscores that combining ultrasonic activation and heating further improves cleaning efficiency, supporting its potential clinical application as an alternative to conventional irrigation protocols.

While our study utilized conventional Scanning Electron Microscopy (SEM) to evaluate smear layer removal, we recognize the potential benefits of employing advanced techniques such as Energy‐Dispersive X‐ray Spectroscopy (EDX) and Environmental Scanning Electron Microscopy (ESEM). These methods enable detailed analysis of the elemental composition of the smear layer and residual debris, facilitating the detection of organic tissues and trace elements. For instance, a study by Prati et al. (2020) utilized ESEM‐EDX to assess the morphology and composition of remnants after retreatment procedures, highlighting the capability of these techniques to provide in‐depth compositional data. Incorporating EDX and ESEM analyses in future research could enhance our understanding of the chemical nature of the smear layer and the efficacy of various irrigation protocols in its removal.

## 5 Study Limitations

This study presents several limitations that should be acknowledged. First, the use of a single tooth for the Micro‐CT analysis, although it enabled standardized comparisons of irrigant penetration across different protocols within identical anatomical conditions, does not reflect the inherent variability among teeth. Anatomical differences can significantly influence irrigant flow and penetration, thereby limiting the generalizability of the results.

Second, the radiopaque contrast medium Hypaque was used in place of sodium hypochlorite (NaOCl) to visualize irrigant penetration via Micro‐CT. While Hypaque replicates important physical characteristics of NaOCl—such as surface tension, viscosity, and specific gravity—it lacks its chemical properties, including antimicrobial activity and tissue dissolution capacity. This methodological choice may affect the interpretation of results in terms of biological performance.

Third, the study focused exclusively on single‐rooted teeth with minimal curvature (less than 10°). Although this approach allowed for greater experimental control, it does not represent the complexity of clinical situations involving multi‐rooted, curved, or anatomically irregular canals. Future research should explore the behavior of irrigants under more challenging anatomical conditions and validate these findings through in vivo clinical studies.

Furthermore, this study focused exclusively on the mechanical aspects of irrigation efficacy, namely the removal of debris and smear layer. However, a true evaluation of root canal disinfection should also consider microbiological factors. The absence of microbial analysis—such as PCR, next‐generation sequencing (NGS), or confocal microscopy for biofilm detection—represents an additional limitation. Future studies should integrate microbiological assessments to better understand the antimicrobial performance of the evaluated irrigation protocols.

## 6 Limitations of the 3D Cleaning Technique

Despite its demonstrated advantages, the 3D Cleaning technique presents several limitations that warrant consideration:

**Limited Applicability in Long and Curved Canals:** The heating plugger may have restricted access to the apical third in anatomically complex canals, particularly those that are long and severely curved. This limitation may compromise the effectiveness of the smear layer and debris removal in these regions, potentially affecting the overall debridement quality.

**Risk of Thermal Damage to Periodontal Tissues:** There is concern regarding potential thermal injury to the periodontal ligament and surrounding structures. This risk may arise from

- **Direct Contact:** Accidental contact between the heated plugger and canal walls in proximity to the apex may result in localized temperature elevation and subsequent tissue damage.
- **Indirect Heat Transfer:** Prolonged activation or the use of elevated temperature settings can lead to thermal conduction through the dentinal walls, increasing the risk of exceeding the physiological temperature threshold for periodontal safety.

These limitations underscore the importance of following strict clinical protocols, including careful temperature control, limiting activation time, and selecting pluggers with appropriate flexibility to navigate complex root canal anatomies.

## 7 Clinical Implications

The findings suggest that advanced irrigation techniques, particularly ultrasonic activation combined with heating, can enhance root canal cleanliness. This method may contribute to higher treatment success rates by improving cleaning efficiency and irrigant penetration, especially in challenging apical regions.

## 8 Conclusion

This study demonstrated that ultrasonic activation combined with internal heating significantly improved the removal of debris and smear layer compared to traditional syringe irrigation and other activation techniques. Additionally, it enhanced irrigant penetration, especially in the apical third, where conventional irrigation methods are often inadequate. These findings support the adoption of advanced irrigant activation techniques to improve root canal disinfection and clinical outcomes. Further studies, particularly in vivo investigations, are needed to validate the long‐term benefits of this approach in complex root canal anatomies.

## Author Contributions

We, the undersigned authors, hereby declare the following: All authors have significantly contributed to the conception, design, execution, analysis, and/or interpretation of the study, as well as to the drafting or critical revision of the manuscript. Each author has played a substantial role in the work, ensuring its intellectual content and quality. All authors have reviewed the final version of the manuscript and agree with its content. Furthermore, all authors consent to its submission for publication in its current form.

## Ethics Statement

The study was conducted according to the guidelines of the Declaration of Helsinki and approved by the Institutional Review Board: protocol code CE‐2025‐16, University of Strasbourg, France.

## Consent

Each subject participated voluntarily after signing an informed consent.

## Conflicts of Interest

The authors declare no conflicts of interest.

## Data Availability

The data that support the findings of this study are available on request from the corresponding author. The data are not publicly available due to privacy or ethical restrictions. No datasets were generated for public use during the current study.
